# Anatomy, Etiology, Management, and Medico-Legal Implications of Botulinum-induced Blepharoptosis

**DOI:** 10.2174/0127724328310459240809073519

**Published:** 2024-08-16

**Authors:** Giulio Nittari, Demetris Savva, Filippo Gibelli, Diana Vulcanescu, Domenico De Leo, Giovanna Ricci

**Affiliations:** 1 Telemedicine and Telepharmacy Centre, School of Medicinal and Health Products Sciences, University of Camerino, Camerino, 62032, Italy;; 2 Plastic, Reconstructive and Aesthetic Surgery, Nicosia General Hospital, Nicosia, 2031, Cyprus;; 3 Section of Legal Medicine, School of Law, University of Camerino, Camerino, 62032, Italy;; 4 General Medicine, Nicosia General Hospital, Nicosia 2031, Cyprus;; 5 Department of Diagnostic and Public Health, Section of Forensic Medicine, University of Verona, Verona, 36134, Italy

**Keywords:** Botulinum, blepharoptosis, anatomy, clinical dermatology, medicolegal, axillary hyperhidrosis

## Abstract

Botulinum toxin injections, a popular aesthetic treatment, have over 7.4 million beneficiaries in the U.S. Despite their safety record, these injections pose potential complications. It is essential for aesthetic practitioners to manage these complications with the least impact on the patient. Upper eyelid ptosis, though rare, is a significant side effect of botulinum toxin injections. Through our study, we have identified the etiology, anatomy, and therapeutic management of botulinum-induced blepharoptosis. Hence, the goal of this study was to identify the basic aetiology of blepharoptosis and manage this complication, as well as discuss the basis of medico-legal implications involving this popular drug. The complex medico-legal implications of botulinum toxin-induced blepharoptosis call for continuous discourse, education, and clarity on drug-use legal standards. With evolving global and Italian legislation, practitioners must ensure they meet care standards, weighing treatment benefits against potential legal and ethical outcomes. Blepharoptosis is a rare but significant complication of botulinum-type injections. Etiology and thorough anatomy are crucial for avoiding this complication and handling it with the least impact on the patient. Medico-legal implications are currently not fully established, but the basis of aesthetic treatment standards, as well as continuing medical education, will ensure correct medico-legal coverage of such complications.

## 1 INTRODUCTION

Botulinum toxin injections have emerged as one of the most prevalent aesthetic treatments in today’s world. Currently, over 7.4 million individuals in the United States are benefitting from these injections. On a global scale, the Botox market shows significant growth, with an estimated value of 4.4 billion dollars from a mere 1.3 billion dollars in 2020 [1]. Furthermore, projections indicate that the European botulinum toxin market is predicted to reach a notable estimate of 2.2 million USD by 2028, as per extrapolation statistics. Botulinum toxin is cultivated from the anaerobic, gram-positive bacillus Clostridium botulinum. Eight distinct serotypes (A to H) have been identified so far, with types A and B frequently utilized in medicine to alleviate muscle spasms and manage overactive muscles (botulinum toxin serotype-A is indicated with BoNT-A).

The usage of Clostridium botulinum toxin type A injections for neuromuscular disorders was first authorized in Europe in 1994 [2]. Allergan Pharmaceuticals received a license in 2002 for the usage of Botox in the treatment of axillary hyperhidrosis [3]. The US Food and Drug Administration (FDA) also approved Botox in the same year for addressing moderate to severe glabellar frown lines. Since then, the FDA has endorsed several other applications of botulinum toxin, and a myriad of off-label uses have rendered it a globally preferred treatment [4].

The botulinum toxin, composed of a heavy and light chain, achieves its therapeutic effect by cleaving SNARE proteins essential for nerve activation. Upon injection, the heavy chain triggers the toxin’s endocytosis into presynaptic neurons. The light chain then separates, acting on SNARE proteins to inhibit neurotransmitter release from axonal endings, resulting in muscle paralysis. This effect is temporary as the toxin's activity diminishes, and the cell regenerates the SNARE protein [5].

While botulinum toxin is safer than other injectables, it can still cause complications. Aesthetic practitioners must be skilled in managing these, minimizing patient impact. A deep understanding of anatomy is crucial for prevention and management, especially for rare but significant side effects like blepharoptosis [6].

## 2 METHODS

The number of cases reported in the literature is very limited, which is why we decided to use a case observed by one of the authors in clinical practice as a basis to describe the management of this rare but possible complication [7-17].

A 67-year-old woman presented at our clinic for botulinum toxin treatment of the upper face, specifically the forehead, glabella, and crow’s feet dynamic wrinkles bilaterally. Upon medical history taking, the patient did not mention any medical conditions or previous surgeries, and no allergies were mentioned.

A total of 160 units of Dysport^®^ were used to treat the forehead, glabella, and crow’s feet bilaterally.

After 14 days, the patient returned for a follow-up consultation. At that time, another 20 units of Dysport^®^ were injected into each orbicularis oculi upper portion in an attempt to induce a more prominent brow lift.

Eight days after the second injection treatment, the patient complained of fatigue and heaviness around her left eye. Upon examination, left upper eyelid ptosis was diagnosed. Frontalis muscle activity was still present. The patient at this examination setting mentioned that she had a surgical blepharoplasty 17 years ago and that she has hypothyroidism, which she failed to mention in the initial consultation.

She was prescribed Apraclonidine Hydrochloride 0.5% eye drops and was instructed to apply 1-2 drops to the affected eye three times per day.

Five days after having been following the above-mentioned therapy, the patient reported the beginning of the resolution of her symptoms, and on the seventh day, she stopped using the drops on her own accord.

## 3 DISCUSSION (ANALYSIS)

### 3.1 Botulinum Toxin in the Treatment of Forehead Wrinkles and Blepharospasm: Anatomical Highlights

**Fig. (1):**
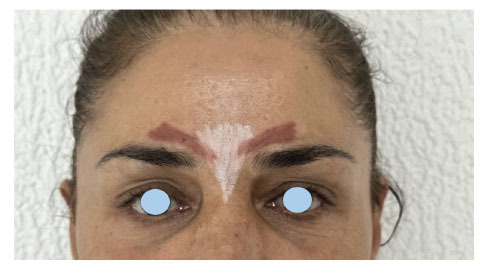
Anatomical position of muscles injected with botulinum toxin for frown lines (procerus-white- and corrugator supercilii muscles-brown).

Upper eyelid elevation is managed through the contraction of the levator palpebrae superioris muscle and Muller’s muscle (superior tarsal muscle). Therefore, we can conclude that the inactivation of either of these muscles by botulinum toxin can result in upper eyebrow ptosis during injection of neighboring structures (Fig. **1**).

The levator palpebrae superioris muscle is a triangular muscle responsible for retraction and elevation of the upper eyelid. It originates from the periosteum of the lesser wing of the sphenoidal bone and proceeds to become the levator aponeurosis with multiple insertions to the upper tarsal plate, upper eyelid skin, and indirectly to the superior conjunctival fornix. Innervation of the levator palpebrae superioris muscle is by the III cranial or ophthalmic nerve [18].

The Muller’s muscle, under the levator palpebrae superioris, comprises smooth fibers attached to the upper eyelid’s superior tarsal plate. It assists the levator muscle, adding 2 mm to eyelid elevation. It is innervated by sympathetic fibers from the superior cervical ganglion [19].

Even if the injector is experienced, there is no way to predict the diffusion of the toxin to the above muscles. Factors like muscle size, anatomic variations of muscles, needle gauge, speed of injection, dilution of toxin, and pressure applied during an injection can play a significant role in the etiology of blepharoptosis as well as underlying conditions like previous facial surgery, myasthenia gravis, Bell’s palsy, and multiple sclerosis [20].

### 3.2 Framing the Complication: Blepharoptosis

Blepharoptosis, more commonly referred to as upper eyelid ptosis, is a medical condition characterized by the drooping or falling of the upper eyelid, a portion of which can cover the eye. This drooping is typically beyond the natural positioning, *i.e*., about 1.5-2 mm below the limbus [21]. The condition can affect one or both eyes and can lead to vision obstruction.

Ptosis can be segmented into five distinct subcategories, each with its unique causes and characteristics [22]:

1. *Myogenic Ptosis*. This subclass is associated with a dysfunction in the muscles responsible for lifting the eyelid, mainly the levator palpebrae superioris. It is commonly seen in conditions like myasthenia gravis and congenital ptosis.
2. *Aponeurotic Ptosis*. Often associated with aging, this form of ptosis results from a weakening or disinsertion of the levator aponeurosis. It is the most common type of acquired ptosis.
3. *Mechanical Ptosis*. This type of ptosis is caused by the weight of excessive skin and fat in the upper eyelid or tumors that pull the eyelid downwards.
4. *Neurogenic Ptosis*. It occurs due to a disruption in the nerve supply to the eyelid muscles. Conditions like Horner’s syndrome, third nerve palsy, and Marcus Gunn's jaw-winking syndrome are typical examples of neurogenic ptosis.
5. *Traumatic Ptosis*. As the name suggests, this form of ptosis results from an injury or trauma to the eye, which can damage the muscles or nerves controlling the eyelid.

The clinical features of botulinum-toxin-induced blepharoptosis vary widely, with onset, duration, spontaneous resolution rates, visual impairment rates, temporal or permanent nature, examination methods, and severity determination varying from case to case [20].

BoNT-A-induced blepharoptosis typically presents within two weeks after the injection. However, the onset can be as early as a few days and as late as a few weeks post-injection. This variation in onset time is due to differences in individual responses to the toxin, diffusion of the toxin, and injection techniques [20-23].

The duration of blepharoptosis varies, but symptomatic relief typically peaks around two weeks post-treatment and may last for three to four months. It is important to note that these are average figures, and the actual duration can be shorter or longer depending on individual factors like the patient’s health status, age, and the dose of the toxin used.

In terms of spontaneous resolution, the majority of cases tend to resolve without any intervention, typically within the period of the toxin’s effect, which is around three to four months. However, a small percentage of cases may require therapeutic interventions.

Regarding visual impairment, while blepharoptosis can cause cosmetic concern and minor discomfort, severe cases can limit vision. The percentage of cases with vision limitation is relatively small compared to those with no vision impairment [24].

When it comes to distinguishing between temporary and permanent ptosis, most cases of BoNT-A-induced blepharoptosis are temporary. Permanent blepharoptosis is extremely rare and is typically associated with repeated high-dose injections or underlying neuromuscular conditions [20].

Clinical examination of blepharoptosis involves measuring the vertical height of the palpebral fissure and the levator function, which is the ability of the levator muscle to lift the eyelid. Severity is typically determined by the degree of drooping and the impact on the visual field.

## 4 DISCUSSION

Upper eyelid ptosis, or blepharoptosis, is the most frequently encountered complication following botulinum toxin injections in the glabellar region. As we previously discussed, the cause of upper blepharoptosis is multifactorial, and several situations can lead to the toxin spreading to neighboring muscles, specifically the levator palpebrae superioris and the superior tarsal muscles.

A thorough understanding of anatomy is vital in minimizing this complication, yet even with such knowledge, anatomical variations or other factors may still result in upper eyelid ptosis, even under the care of the most seasoned injector. It is crucial for the physician to be equipped to manage such complications with minimal disruption to the patient.

To mitigate the risk of eyebrow ptosis, several strategies have been outlined in the medical literature [20-25].

**Fig. (2):**
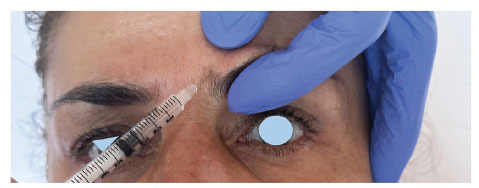
Correct direction and placement of botulinum toxin when injecting corrugator supercilii muscle.

Injections in the glabellar region should be performed 1 cm above the eyebrow, and injections beyond the mid-pupillary line should be avoided. Digital pressure should be applied to the supraorbital rim during injection to avoid diffusion of the toxin, and the needle should be pointing superiorly to avoid the orbital region. Also, slow injection of solution plays a deterrent factor in the chances of eyebrow ptosis (Fig. **2**).

Anatomical variations of vessels in the area of injecting can lead to serious complications. It is important for an injector to know the possible anatomic variations when treating an area to avoid as many as possible complications. For example, bifurcation of the supraorbital artery, in some cases, might cause an injury to the vessel wall with hematoma formation or bruising [26-28].

If blepharoptosis occurs early, a couple of days post injection, it will probably last throughout the whole effect of the toxin (4-6 months). If ptosis occurs later, after a week to 10 days, most probably the results will be transient, lasting about a month due to the diffusion of the toxin slightly to adjacent areas and not actually within the muscles.

Apraclonidine hydrochloride 0.5% is the principal treatment modality for eyebrow ptosis. Apraclonidine hydrochloride is an alpha 2-adrenergic agonist instilled in the conjunctival sac that stimulates the contraction of the Muller’s muscle, causing a transient lid lift of 1-2 mm, compensating in this way the toxin-induced ptosis [29, 30]. Contraindications of apraclonidine eyedrops include pregnancy and nursing, coronary insufficiency, and thromboangiitis obliterans [31-33]. Alternatively, phenylephrine hydrochloride 2.5% can be used, an alpha 1-adreneregic agonist, with its own contraindications of narrow-angle glaucoma due to its mydriatic effect, coronary heart disease, and overactive thyroid gland [34].

## 5 MEDICO-LEGAL IMPLICATIONS

The predictability of blepharoptosis induced by BoNT-A is somewhat controversial. While BoNT-A is known to potentially weaken the levator palpebrae superioris muscle and cause eyelid ptosis, the occurrence is not guaranteed. This is due to the multifactorial nature of the complication, as mentioned above. Factors, such as anatomical variations, incorrect injection techniques, and the diffusion of toxin to adjacent muscles can contribute to the development of ptosis. This unpredictability complicates the legal aspect as it may be challenging to prove the practitioner’s negligence or fault in causing the complication. Preventability, on the other hand, is more straightforward. An in-depth understanding of the patient’s anatomy, careful administration of BoNT-A, and adherence to established injection guidelines can significantly decrease the risk of blepharoptosis. Therefore, any deviation from these practices could potentially lead to malpractice claims.

In Italy, as in most jurisdictions, the standard for malpractice involves proving that the practitioner failed to adhere to the standard of care, which resulted in harm to the patient. In the context of BoNT-A-induced blepharoptosis, a malpractice claim could potentially be successful if it can be proven that the practitioner did not follow the appropriate guidelines or was not adequately cautious during administration, leading to the complication. However, proving malpractice in such cases can be challenging. Given the temporary nature of BoNT-A-induced blepharoptosis, which usually resolves on its own or can be treated with interventions, such as eye drops or additional BoNT-A injections, it may be difficult to demonstrate that the patient suffered substantial harm. Permanent damage, while rare, may offer a stronger case for malpractice. However, the burden of proof remains high, requiring clear evidence that the practitioner’s negligence directly led to the permanent damage.

In response to these challenges, practitioners may resort to defensive medicine, conducting additional tests or administering treatments not necessarily for the patient’s benefit but to protect themselves against potential legal actions. While this may reduce the risk of malpractice claims, it poses ethical concerns and can lead to unnecessary healthcare costs.

Nonetheless, there are other strategies that can reduce the risk of complications and also the emergence of medical-legal disputes.

In other words, there are several strategies that practitioners can employ to mitigate these risks and improve patient outcomes. These strategies go beyond the basics of understanding patient anatomy and adhering to injection guidelines, and they delve into areas, such as patient education, informed consent, continuous learning, and the use of advanced techniques and technologies.

Firstly, patient education plays a crucial role in managing the medico-legal risks associated with BoNT-A injections. Patients must be adequately informed about the potential risks and benefits of the treatment, including the possibility of complications such as blepharoptosis. They should also be made aware of the temporary nature of the complication and the available treatment options. This not only helps manage patient expectations but also contributes to building a strong doctor-patient relationship, which can be instrumental in avoiding legal disputes.

Informed consent is another critical component of managing medico-legal risks. It is not enough to merely obtain a signed consent form from the patient. Practitioners should ensure that the consent process is thorough, with the patient demonstrating a clear understanding of the treatment, its potential complications, and the proposed plan for managing any adverse outcomes. Documentation of this process is equally important, as it provides a record that the patient was fully informed and willingly consented to the treatment [35-38].

Continuing education is another important strategy. The realm of aesthetic dermatology is continually evolving, with new techniques, technologies, and research findings emerging regularly. By staying abreast of the latest developments, practitioners can refine their techniques, improve patient safety, and reduce the likelihood of complications. This not only enhances patient outcomes but also bolsters the practitioner’s defense in the event of a malpractice claim [39, 40].

Technological advancements can also contribute to mitigating medico-legal risks. For example, ultrasound-guided injections have been shown to improve the precision of BoNT-A administration, reducing the risk of unintended diffusion to adjacent muscles [41].

Finally, practitioners should consider the potential benefits of professional liability insurance. This type of insurance can provide crucial financial protection in the event of a malpractice claim, helping to cover the costs of legal defense and any damages awarded to the patient. However, it is essential to fully understand the terms of the insurance policy, including what is covered and what is not.

## CONCLUSION

While Botulinum toxin injections are considered generally safe injectables, complications and side effects can occur. To minimize these risks, practitioners must have a very good understanding of anatomy, take detailed patient history, and exhibit precise injection techniques.

However, if complications like blepharoptosis do occur, clinicians should be able to manage them with minimal impact on the patient. The complexities of botulinum-induced blepharoptosis have medical-legal implications, emphasizing the importance of education and legal clarity, both in Italy and globally. With growing popularity and evolving laws, practitioners must balance treatment benefits, and potential legal consequences, maintaining the standard of care always for the benefit of the patient.

Mastering the anatomy, etiology, and management of this treatment through a good theoretical analysis gives the injector the competence to perform this treatment and, in case of such a complication, to be able to treat it with the least implication on the patient as well as himself regarding the medicolegal aspects.

## AUTHORS' CONTRIBUTIONS

D.S., as the first author and the originator of the article idea, took the lead in the conceptualization and analysis of clinical data, as well as in drafting the initial manuscript. He was deeply involved with the patient’s care and follow-up. He provided critical clinical insights and was instrumental in shaping the manuscript’s overall narrative and discussion.

G.N., D.V., and G.R. contributed to the literature review, manuscript methodology, and editing. They played key roles in ensuring the manuscript’s scientific rigor and clarity of communication.

F.G. and D.D.L., both forensic physicians, were chiefly responsible for the exploration and description of the medico-legal implications of the case. Their expertise ensured a thorough and nuanced understanding of these complex issues.

Finally, D.D.L., the last author, supervised the whole project. He provided strategic oversight and intellectual guidance, ensuring the coherence and integrity of the study.

Each author approved the final version of the manuscript and agreed to be accountable for all aspects of the work. All authors met the journal’s criteria for authorship.

## ABBREVIATION

FDA: US Food and Drug Administration
